# Toxicological insight of metiram: immuno-oxidative, neuro-behavioral, and hemato-biochemical changes during acute exposure of Nile tilapia (*Oreochromis niloticus*)

**DOI:** 10.1186/s12917-024-04126-4

**Published:** 2024-07-09

**Authors:** Mohamed Shaalan, Mohamed A. Elbealy, Mahmoud I. M. Darwish, Elsayed M. Younis, Abdelwahab A. Abdelwarith, Asmaa I. Abdelaty, Simon J. Davies, Rowida E. Ibrahim, Afaf N. Abdel Rahman

**Affiliations:** 1 Department of Pathology, Faculty of Veterinary Medicine, Cairo University, PO Box 12211, Giza, Egypt; 2 Department of Aquatic Animal Medicine, Faculty of Veterinary Medicine, Mansoura University, PO Box 35516, Mansoura, Dakahlia, Egypt; 3 Department of Biochemistry and Molecular Biology, Medicine Faculty of Veterinary Medicine, Zagazig University, PO Box 44511, Zagazig, Egypt; 4 Department of Zoology, College of Science, King Saud University, PO Box 2455, Riyadh, 11451 Saudi Arabia; 5 Department of Behaviour and Management of Animal, Poultry and Aquatics, Faculty of Veterinary Medicine, Zagazig University, PO Box 44511, Zagazig, Egypt; 6 Aquaculture Nutrition Research Unit ANRU, Ryan Institute, College of Science and Engineering, Carna Research Station, University of Galway, Galway, H91V8Y1 Ireland; 7 Department of Aquatic Animal Medicine, Faculty of Veterinary Medicine, Zagazig University, PO Box 44511, Zagazig, Egypt; 8 Polymer Institute, Slovak academy of sciences, Dúbravská cesta 9, Bratislava, 84541 Slovakia

**Keywords:** Acute exposure, Aquatic toxicology, Behaviour, Blood picture, *Oreochromis niloticus*, Polyram DF

## Abstract

**Background:**

The inappropriate use of pesticides including fungicides creates severe biological hazards that can endanger fish health and impede sustainable aquaculture.

**Objective:**

This study investigated the negative impacts of metiram (MET), a fungicide on the health status of Nile tilapia (*Oreochromis niloticus*) for a 96-hour duration as an acute exposure in a static renewal system.

**Methods:**

Three hundred fish (average body weight: 37.50 ± 0.22 g) were assigned into six groups (50 fish/group) with five replicates (10 fish/replicate). Fish were exposed to various six concentrations (0, 1.5, 3, 4.5, 6, and 7.5 mg/L) of MET as a water exposure to for 96-hour without water exchange. The fish’s behavior, clinical signs, and mortalities were documented every day of the exposure period. Additionally, MET’s impact on blood profile, stress biomarkers, hepato-renal functions, immune-antioxidant status, and brain biomarker were closely monitored.

**Results:**

The lethal concentration (LC_50_) of MET estimated using Finney’s probit technique was 3.77 mg/L. The fish’s behavior was severely impacted by acute MET exposure, as clear by an increase in surfacing, loss of equilibrium, unusual swimming, laterality, abnormal movement, and a decline in aggressive behaviors. The survivability and hematological indices (white and red blood cell count, differential white blood cell count, hematocrit value, and hemoglobin) were significantly reduced in a concentration-dependent manner following MET exposure. Acute exposure to MET (1.5–7.5 mg/L) incrementally increased stress biomarkers (nor-epinephrine, cortisol, and glucose), lipid peroxides (malondialdehyde), and brain oxidative DNA damage biomarker (8-hydroxy-2-deoxyguanosine). A hepato-renal dysfunction by MET exposure (4.5–7.5 mg/L) was evidenced by the significant increase in the alanine and aspartate aminotransferases and creatinine values. Moreover, a substantial decline in the immune parameters (lysozyme, complement 3, serum bactericidal activity, and antiprotease activity) and antioxidant variables (total antioxidant capacity, superoxide dismutase, and glutathione peroxidase) resulted from acute MET exposure.

**Conclusion:**

According to these findings, the 96-hour LC_50_ of MET in Nile tilapia was 3.77 mg/L. MET exposure triggered toxicity in Nile tilapia, as seen by alterations in fish neuro-behaviors, immune-antioxidant status, hepato-renal functioning, and signifying physiological disturbances. This study emphasizes the potential ecological dangers provoked by MET as an environmental contaminant to aquatic systems. However, the long-term MET exposure is still needed to be investigated.

## Introduction

Pollution is a major concern that affects everyone, and it is being exacerbated by the world’s industrialization and expanding population. Environmental pollution caused by chemical abuse poses potential risks to all levels of biological organization, including aquatic species [1, 2]. A wide range of harmful pollutants, such as agrochemicals and pesticides have been introduced into the ecosystem through wash-off, irrigation, and drift as a result of development processes and agricultural activities [3]. Pesticides are chemicals for managing pests and boosting the output of crops. They can contaminate feed ingredients and the aquatic environment, posing serious risks to fish and other creatures [4]. The possibility for these pesticides in water to bioaccumulate in species other than the intended target (fish tissues) as well as strengthen at higher levels throughout the food chain presents a risk. These pesticides may negatively impact aquatic biota diversity and water habitats [5, 6].

Fungicides, a type of pesticides, are widely used to combat a wide range of fungal infections in various field crops and fruits [7]. Some fungicides are hazardous to humans, animals, and important plants due to their persistence in the environment [8]. Among agricultural fungicides, metriram (MET) is an ethylenebisdithiocarbamates-based fungicide (EBDCs) and non-cholinesterase inhibiting that acts non-systemically [9]. MET (zinc; N-[2-(sulfidocarbothioylamino)ethyl]carbamodithioate) has been used to manage fungal diseases such as brown and black spots and early blight on a wide range of vegetables, fruits, and ornamental crops for over 40 years [10].

Breakdown products of MET are ethylene thiourea (ETU), propylene thiourea (PTU), and carbon disulfide have been reported to induce teratogenicity, neurotoxicity, immunotoxicity, carcinogenicity, and anti-thyroid potential in rats [11, 12]. Prior research on human cells has indicated that MET can induce cytotoxicity and damage to DNA [13]. MET can reach surface waterways unexpectedly for instance through spray drift. MET can be treated regularly (three to nine times) in crop protection programs, ultimately leading to recurrent short-term exposures in surface water of roughly 0.28–25 µg/L [14]. MET was detected in mushroom samples at a detection limit of 0.05 mg/kg [15].

Fish is an effective model for measuring pesticide toxicity in aquatic environments due to its high pesticide sensitivity, ability to metabolize pollutants, and bioaccumulation [16, 17]. The *Oreochromis niloticus* L. (Nile tilapia) is a popular aquaculture fish due to its suitability for aquaculture, rapid growth, and high palatability [18]. Nile tilapia is frequently employed as a biological indicator of environmental pollution and for evaluating the quality of the aquatic environment [19]. Pesticide exposure resulted in several toxicologically negative consequences on the metabolic and biological systems of fish (non-target species) [20]. Acute toxicity results of MET show that the lethal concentration 50 (LC_50_) values are 333 µg/L to less than 20,000 µg/L for fish, 110 µg/L to less than 1,000 µg/L for aquatic invertebrates, and 63 µg/L to less than 1,000 µg/L for algae [14]. MET had severe toxic impacts on zebrafish (*Danio rerio*) embryos including immune-antioxidant alterations and endocrine disruption [21]. Also, several fish species, including rainbow trout (*Oncorhynchus mykiss*), common carp (*Cyprinus carpio*), sheepshead minnow (*Cyprinodon variegatus*), and bluegill (*Lepomis macrochirus*) were subjected to acute MET toxicological studies [22].

Nevertheless, information regarding the harmful effects of MET on Nile tilapia and possible pathways is still lacking. Due to the paucity of information is in this field. The objective of this work was to determine 96-hour LC_50_ of MET in Nile tilapia. We also intended to look into how fish’s neuro-behavioral, hemato-immunological, antioxidant, and stress responses were affected by a 96-hour acute exposure to various MET concentrations.

## Materials and methods

### MET preparation and animal ethics

For this investigation, MET was purchased from BASF SE Company (Ludwigshafen, Germany) as Polyram® DF 80%. To create a stock solution, distilled water was used to dissolve MET. The Zagazig University Authority for Animal Use in Research gave its approval for this work (ZU-IACUC/2/F/3/2024).

### Fish culturing conditions

Nile tilapia (average body weight, 37.50 ± 0.22 g) were gathered from the Kafr ELSheikh Governorate fish farm in Egypt. The fish were examined in great detail in accordance with CCAC [23] recommendations to ascertain their health. Ten fish were placed in an 80 × 70 × 35 cm indoor glass tank with good ventilation. Fish tanks contained dechlorinated tap water and were connected to a central air compressor and for air stones constant aeration. Before the study, fish were acclimated for 15 days, receiving a basal diet three times a day (9:00, 13:00, and 17:00 h) until they reached satiation.

Throughout the trial and acclimation periods, water quality variables were tracked and kept within allowable bounds following the APHA [24] guidelines. The pH (6.4 ± 0.20), dissolved oxygen (6.73 ± 0.28 mg/L), temperature (23.00 ± 1.20 °C), nitrite (0.03 ± 0.01 mg/L), and ammonia (0.01 ± 0.003 mg/L) were among these variables. Two times a week, the entire water was replaced, and any excrement from the tank bottom was emptied via daily siphoning during the acclimation time.

### Experimental design and behavioral observation

Fish were randomly split into six groups, each with five replicates (50 fish per group; 10 fish per replicate), and exposed to varying MET concentrations (0, 1.5, 3, 4.5, 6, and 7.5 mg/L) as a water exposure for 96-hour. Mortalities were recorded twice a day to ascertain the 96-hour LC_50_ of the fungicide under investigation using a probit analysis program. Throughout the trial, fish were checked daily for 96-hour to collect data and record the clinical symptoms.

Following the protocol of Altmann [25], fish behaviors were monitored using an adjustable timer camera via the scan sampling approach. Throughout the 96-hour trial, the behavioral patterns were monitored for 5 min/aquarium twice per day. The frequencies of behaviors were computed. The monitored behaviors were surfacing and unusual swimming [26], loss of equilibrium [27], resting [28], laterality [29], and abnormal movement [30]. Moreover, aggressive behaviours (spreading of fin, approach, and mouth pushing) were also monitored [31].

### Sampling

By the end of the trial (96-hour), the fish was sedated (100 mg/L benzocaine solution) following a prior method [32]. Twelve randomly chosen fish per group were used for blood collection from caudal blood vessels. Two sets of blood samples were gathered; one of them was taken in a 1 mL heparinized syringe for hematological investigation. The other set was aspirated using 1 mL of the anticoagulant-free plastic syringe and kept at 4 °C to coagulate overnight. These samples were centrifuged at 1750 *x*g for 10 min to extract the serum for biochemical and immunological assays. Moreover, benzocaine solution (300 mg/L) was used to euthanize the fish [33]. Samples of the liver and brain (12 fish/group) were taken to assess hepatic antioxidant/oxidant and neuro-related biomarkers.

### Hematological and stress-related assays

Using the automated hematology analyzer from Hospitex Diagnostics (Sesto Fiorentino, Italy), the hematological variables were analyzed using a previous method [34]. These metrics comprised red blood cells (RBCs), hematocrit value (Hct %), and hemoglobin (Hb) concentration. Also, the assessment included total leucocyte count (WBCs) and their differential counts. The mean corpuscular hemoglobin (MCH), MCH concentration (MCHC), and mean corpuscular volume (MCV) were estimated following these formulas:

$$\text{M}\text{C}\text{H}= 10 \times \frac{\text{H}\text{b}}{\text{R}\text{B}\text{C}\text{s}}$$

$$\text{M}\text{C}\text{H}\text{C}= 100 \times \frac{\text{H}\text{b}}{\text{H}\text{c}\text{t}}$$

$$\text{M}\text{C}\text{V}= 10 \times \frac{\text{H}\text{c}\text{t}}{\text{R}\text{B}\text{C}\text{s}}$$

Moreover, serum samples were used to estimate the level of nor-epinephrine (Cat No.: MBS025809) and cortisol (Cat No.: MBS704055) hormones using the ELISA commercial kits (My-Biosource Inc., San Diego, California, USA). Using a method of Trinder [35], the serum glucose (GLU; Cat No.: GL 1320) level was assessed spectrophotometrically using commercial kits of a Bio diagnostics company.

### Biochemical and immunological assays

Commercial kits of Biodiagnostic Co. (Egypt) Cat. No. AL 1031, AS 1061, and CR 1250 were used for the determination of serum alanine (ALT) and aspartate (AST) aminotransferases and creatinine, respectively, following previously described approaches [36, 37].

Lysozyme (LYZ) activity, complement 3 (C3) level, serum bactericidal activity% (SBA %), and antiprotease activity were evaluated as immunological indicators. The Ellis [38] technique was used for evaluating the serum LYZ activity. After being suspended in 2 mL of 0.05 M sodium phosphate buffer (pH 5.9), approximately 0.25 mg/mL of *Micrococcus lysodeikticus* (Sigma-Aldrich Chemie GmbH, Darmstadt, Germany) was allowed to incubate for 5 min at 30 °C. Following that, 200 µL of serum samples were added, and the absorbance at 450 nm was determined. The C3 level (Cat No.: CSB-E09727s) was quantified by spectrophotometry using Cusabio kits, adhering to the guidelines that came with the kit packing. Moreover, SBA % was calculated using the Wangkaghart et al. [39] procedure against *Streptococcus agalactiae* and displayed as serum bactericidal percentage. To measure the antiprotease activity, the serum samples were also treated for five minutes with 0.1% trypsin (HiMedia) [40].

### Hepatic antioxidant/oxidant and neuro-related assays

The antioxidant variables [total antioxidant capacity (TAC), superoxide dismutase (SOD), and glutathione peroxidase (GPx)] and lipid peroxides biomarker (malondialdehyde; MDA) levels were assayed in liver samples. Following the liver homogenization in buffer (pH 7.4), the resultant homogenates were centrifuged at 10,000 *x*g for 20 min at 4 °C and then centrifuged again at 10,000 g for 1 h at 4 °C. The pellet was washed and preserved in a pH 7.4 buffer [41]. The values of TAC (CAT. No. TA 25 13), SOD (CAT. No. SOD 2521), GPx (CAT. No. GP 2524), and MDA (CAT. No. MAD 25 29) were measured using Bio-diagnostic kits (Egypt) following prior protocols [42–45].

The levels of oxidative DNA damage biomarker (8-hydroxy-2-deoxyguanosine; 8-OHdG) and neurotransmitter (acetylcholine esterase; AchE) in all experimental groups were measured spectrophotometrically using brain tissue samples. Brain samples were homogenized in 150 mM sodium chloride (15 mL) followed by centrifugation at 5 °C (3000 *x*g, 15 min). The level of 8-OHdG was estimated using the kit (My-Biosource Inc., San Diego, California, USA) following the previous method [46]. The AchE activity was measured at 450 nm detection wavelength using a commercial kit (Cat. No. MBS280290) (My-Biosource Inc., San Diego, California, USA) following the previous methodology [47].

### Data analysis

The 96 h-LC_50_ was computed using the probit analysis program (version 1.5, US Environmental Protection Agency). The Kaplan-Meier approach was employed to ascertain the fish survival rate. To seek any differences, the log-rank test was performed in pairwise comparisons. The Shapiro-Wilk test was used to ensure that the gathered data were normal. Following that, a one-way ANOVA (SPSS 20.0, IBM Corp.) was applied to all data that were demonstrated as means ± standard error (*SE*). To look for differences in means at *P* < 0.05, Duncan’s post hoc test was used.

## Results

### The 96-hour LC_50_ of MET, survival rate, behaviors, and clinical observations

**Fig. 1:**
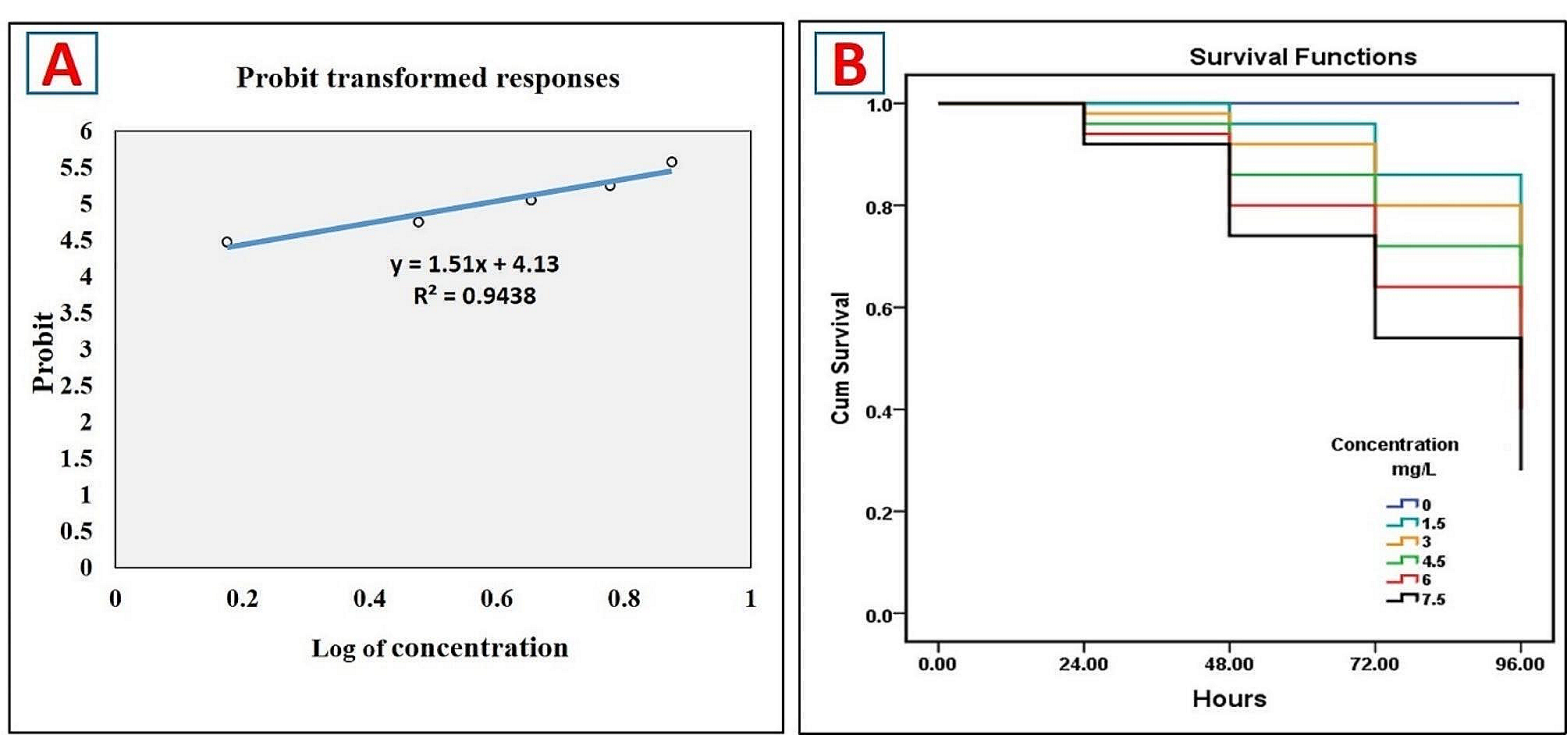
Acute toxicity (96-hour) of metriram (MET) in Nile tilapia. **A** Probit finding to compute 96-hour LC_50_ of MET. **B** Kaplan–Meier survival curves for varying MET concentrations (24–96 h of exposure)

The obtained results (Fig. 1A) from the probit analysis showed that the 96-hour LC_50_ of MET was 3.77 mg/L. In a concentration-dependent way, Fig. 1B illustrates a declining survivability. The survival percentage in 0, 1.5, 3, 4.5, 6, and 7.5 mg/L of MET concentrations was 100, 70, 60, 48, 40, and 28%, based on the Kaplan–Meier curves. Furthermore, statistical significance was observed for the variances among the groups (*P* < 0.001).

The MET exposure had an impact on the behaviors of the fish (Table 1). Acute MET exposure (1.5–7.5 mg/L) significantly (*P* < 0.001) increased the surfacing, unusual swimming, and abnormal movement (spiral and circular) in a level-dependent manner compared to the control group (0 mg/L MET). The loss of equilibrium, laterality, and resting showed substantial elevation (*P* < 0.001) and the aggressive behaviors (spreading of the fin, approach, and mouth pushing) were markedly declined (*P* < 0.001) with acute MET exposure (3–7.5 mg/L) compared with the control.

**Fig. 2:**
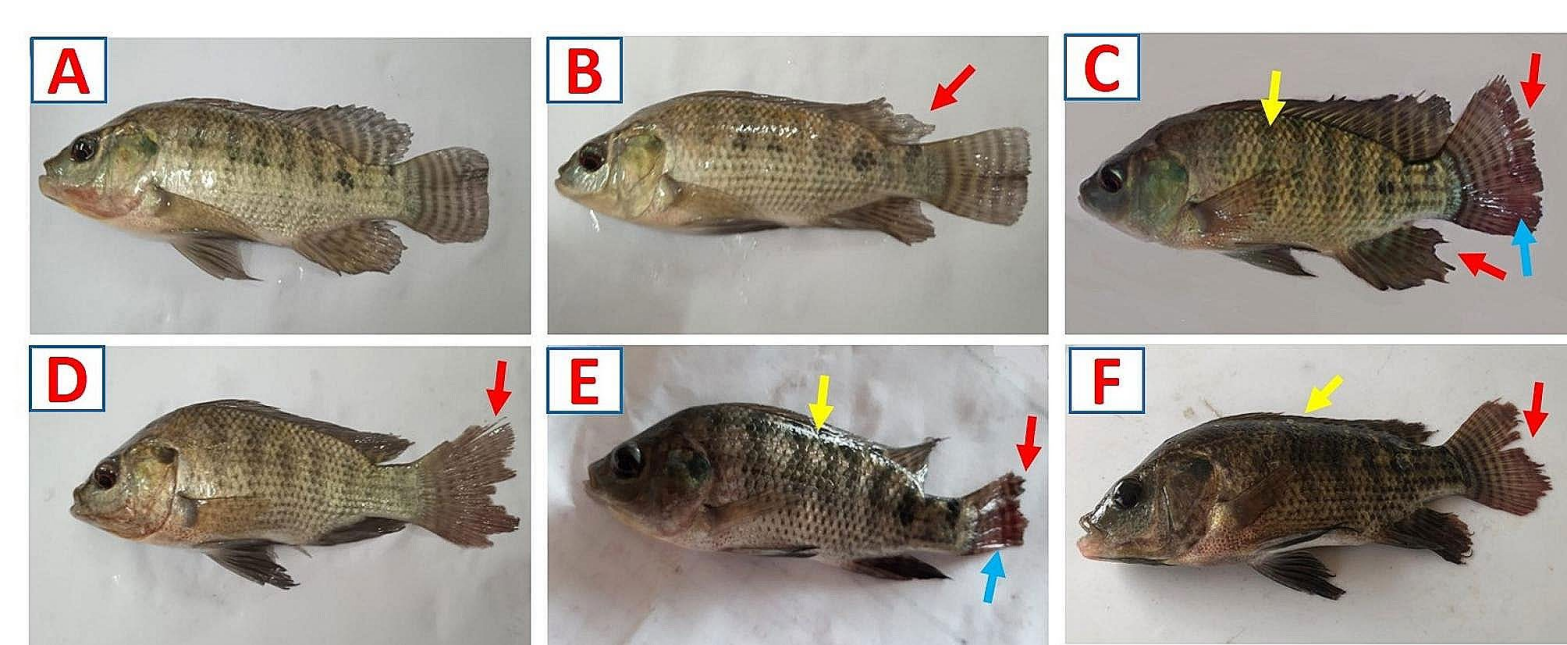
Impact of various concentrations of metriram (MET) exposure for 96-hour on clinical observation in Nile tilapia. **A** Control fish (0 mg/L MET) exhibit a normal appearance. **B − F** Fish that were exposed to 1.5, 3, 4.5, 6, and 7.5 mg/L MET, respectively, exhibit skin darkness (yellow arrows), fin rot (red arrows), and hemorrhages at the caudal fin (light blue arrows)

The non-exposed fish (control) did not reveal any clinical signs (Fig. 2A). In contrast, a rise in MET concentration was associated with a variety of clinical signs (Fig. 2B–F). The MET-exposed fish showed skin darkness, fin rot, and hemorrhages at the caudal fin, which were more noticeable as the concentration of MET increased.

**Table 1:** Impact of various concentrations of metiram (MET) exposure for 96-hour on behaviors of Nile tilapia

Behaviors	Concentration of MET (mg/L)						*P*-value
	0	1.5	3	4.5	6	7.5	
Surfacing	0.25 ± 0.03^f^	0.94 ± 0.02^e^	1.84 ± 0.02^d^	2.14 ± 0.03^c^	3.42 ± 0.01^b^	5.90 ± 0.01^a^	*P* < 0.001
Loss of equilibrium	0.00 ± 0.00^e^	0.04 ± 0.01^e^	3.44 ± 0.02^d^	5.15 ± 0.03^c^	6.36 ± 0.05^b^	9.38 ± 0.05^a^	*P* < 0.001
Unusual swimming	0.14 ± 0.01^f^	1.83 ± 0.02^e^	2.44 ± 0.03^d^	5.45 ± 0.06^c^	7.58 ± 0.08^b^	10.82 ± 0.01^a^	*P* < 0.001
Resting	0.63 ± 0.31 ^e^	0.97 ± 0.67^e^	1.78 ± 0.55^d^	3.93 ± 0.51 ^c^	5.70 ± 0.40^b^	8.52 ± 0.71^a^	*P* < 0.001
Laterality	0.00 ± 0.00^e^	0.01 ± 0.02^e^	2.14 ± 0.03^d^	3.63 ± 0.01^c^	5.32 ± 0.02^b^	7.27 ± 0.03^a^	*P* < 0.001
**Aggressive behaviour**							
Spreading of fin	14.12 ± 0.75^a^	15.98 ± 0.89^a^	12.78 ± 0.45^b^	8.59 ± 0.17^c^	6.69 ± 0.28^d^	5.72 ± 0.41^e^	*P* < 0.001
Approach	10.11 ± 0.69^a^	10.00 ± 0.57^a^	5.71 ± 0.24^b^	4.17 ± 0.16^c^	3.40 ± 0.23^d^	2.90 ± 0.42^e^	*P* < 0.001
Mouth pushing	6.23 ± 0.60^a^	6.00 ± 0.61^a^	4.61 ± 0.36^b^	3.46 ± 0.20^c^	2.36 ± 0.11^d^	1.28 ± 0.27 ^e^	*P* < 0.001
**Abnormal movement**							
Spiral movement	0.00 ± 0.00^f^	2.93 ± 0.03^e^	3.52 ± 0.01^d^	4.39 ± 0.02^c^	5.91 ± 0.01^b^	8.82 ± 0.04^a^	*P* < 0.001
Circular movement	0.00 ± 0.00^f^	1.04 ± 0.07^e^	2.47 ± 0.27^d^	5.00 ± 0.13^c^	6.80 ± 0.38^b^	9.85 ± 0.41^a^	*P* < 0.001

Values (mean ± *SE*) not sharing superscripts in the same row are significantly different (*P* < 0.05; One-way ANOVA)

### Hematological variables

The hematological profile of Nile tilapia subjected to acute MET toxicity is displayed in Table 2. After acute MET exposure (1.5–7.5 mg/L), the total WBCs and their differential count (heterophils, lymphocytes, eosinophils, basophils, and monocytes) revealed a significant decrease (*P* < 0.001) compared with the control group (0 mg/L MET) in a level-dependent way.

A marked reduction (*P* < 0.001) in RBCs count, Hct%, Hb, and MCHC level was obvious by MET acute exposure (1.5–7.5 mg/L) relative to the control group except for MCHC which was unaffected by 1.5 mg/L MET. In contrast, MCH and MCV values showed a significant increase by acute MET exposure of 4.5–7.5 mg/L and 3–7.5 mg/L, respectively. The highest values of MCH and MCV were noted in the 7.5 mg/L concentration.

**Table 2:** Impact of various concentrations of metiram (MET) exposure for 96-hour on hematological variables of Nile tilapia

Parameters	Concentration of MET (mg/L)						*P*-value	
	0	1.5	3	4.5	6	7.5		
**Leukogram**								
WBCs ×10^^3^/cmm	17.90 ± 0.52^a^	14.08 ± 0.04^b^	11.08 ± 0.05^c^	9.77 ± 0.07^d^	8.09 ± 0.05^e^	6.32 ± 0.19^f^	*P* < 0.001	
Heterophils	8.59 ± 0.25^a^	6.75 ± 0.02^b^	5.32 ± 0.02^c^	4.69 ± 0.03^d^	3.88 ± 0.03^e^	3.03 ± 0.09^f^	*P* < 0.001	
Lymphocytes	8.16 ± 0.24 ^a^	6.42 ± 0.02^b^	5.05 ± 0.02^c^	4.45 ± 0.03^d^	3.69 ± 0.02^e^	2.88 ± 0.08^f^	*P* < 0.001	
Eosinophils	0.27 ± 0.01^a^	0.21 ± 0.001^b^	0.16 ± 0.001^c^	0.14 ± 0.001^d^	0.12 ± 0.001^e^	0.09 ± 0.002^f^	*P* < 0.001	
Basophils	0.18 ± 0.01^a^	0.14 ± 0.004 ^b^	0.11 ± 0.003 ^c^	0.09 ± 0.001^d^	0.08 ± 0.001^e^	0.06 ± 0.001^f^	*P* < 0.001	
Monocytes	0.70 ± 0.02^a^	0.55 ± 0.002 ^b^	0.44 ± 0.001^c^	0.39 ± 0.002 ^d^	0.31 ± 0.002^e^	0.25 ± 0.01^f^	*P* < 0.001	
**Erythrogram**								
RBCs ×10^^6^/cmm	5.73 ± 0.08^a^	4.55 ± 0.15^b^	3.66 ± 0.09^c^	2.89 ± 0.05^d^	2.26 ± 0.06^e^	1.45 ± 0.08^f^		*P* < 0.001
Hct (%)	36.48 ± 0.26^a^	28.79 ± 0.05^b^	27.68 ± 0.04^c^	26.62 ± 0.07^d^	23.67 ± 0.02^e^	21.05 ± 0.54^f^		*P* < 0.001
Hb (g/dL)	12.29 ± 0.06^a^	9.52 ± 0.13^b^	8.46 ± 0.05^c^	7.66 ± 0.08^d^	6.28 ± 0.17^e^	5.11 ± 0.06^f^		*P* < 0.001
MCH	21.45 ± 0.39^c^	20.95 ± 0.41^c^	23.13 ± 0.71^c^	26.49 ± 0.24^b^	27.88 ± 1.57^b^	35.31 ± 1.60^a^		*P* < 0.001
MCHC	33.69 ± 0.23^a^	33.05 ± 0.38^a^	30.57 ± 0.14^b^	28.79 ± 0.21^c^	26.53 ± 0.70^d^	24.32 ± 0.93^e^		*P* < 0.001
MCV	63.65 ± 1.13^e^	63.42 ± 1.97^e^	75.64 ± 1.98^d^	92.04 ± 1.30^c^	104.93 ± 3.14^b^	146.08 ± 12.23^a^		*P* < 0.001

Values (mean ± *SE*) not sharing superscripts in the same row are significantly different (*P* < 0.05; One-way ANOVA). WBCs: white blood cells; RBCs: red blood cells; Hct: hematocrit value; Hb: hemoglobin; MCH: mean corpuscular hemoglobin; MCHC: MCH concentration; MCV: mean corpuscular volume

### Stress-related and hepato-renal function variables

The levels of stress-related biomarkers (nor-epinephrine, cortisol, and GLU) of Nile tilapia following acute MET exposure are shown in Table 3. There was a significant increase (*P* < 0.001) in these variables by MET exposure. This elevation was in a manner based on the MET concentration.

The variables of hepato-renal functioning (ALT, AST, and creatinine) following Nile tilapia exposure to acute MET toxicity are displayed in Table 3. The MET exposure (4.5–7.5 mg/L) substantially (*P* < 0.001) elevated the hepatic (ALT and AST) and renal (creatinine) markers. The maximum value was observed at the 7.5 mg/L MET exposure level. MET (1.5 and 3 mg/L) exposure did not alter these parameters.

**Table 3:** Impact of various concentrations of metiram (MET) exposure for 96-hour on stress-related and hepato-renal function variables of Nile tilapia

Parameters	Concentration of MET (mg/L)						*P*-value
	0	1.5	3	4.5	6	7.5	
**Stress-related variables**							
Nor-epinephrine (pg/mL)	0.86 ± 0.02^f^	1.69 ± 0.12^e^	3.55 ± 0.14^d^	6.12 ± 0.05^c^	11.19 ± 0.11^b^	15.40 ± 0.12^a^	*P* < 0.001
Cortisol (ng/mL)	14.41 ± 0.24^f^	37.28 ± 0.16^e^	67.18 ± 0.68^d^	81.47 ± 0.84^c^	106.38 ± 0.79^b^	126.25 ± 0.43^a^	*P* < 0.001
GLU (mg/dL)	72.08 ± 0.66^f^	87.06 ± 0.43^e^	101.55 ± 1.55^d^	116.71 ± 1.49^c^	137.12 ± 1.80^b^	171.81 ± 0.98^a^	*P* < 0.001
**Hepato-renal function variables**							
ALT (U/L)	12.89 ± 0.51^d^	13.19 ± 0.22^d^	13.11 ± 0.06^d^	28.73 ± 0.15^c^	38.01 ± 1.16^b^	50.45 ± 0.31^a^	*P* < 0.001
AST (U/L)	11.45 ± 0.26^d^	12.11 ± 0.13^d^	12.39 ± 0.08^d^	26.19 ± 0.69^c^	37.26 ± 0.73^b^	54.80 ± 0.69^a^	*P* < 0.001
Creatinine (mg/dL)	0.30 ± 0.01^d^	0.31 ± 0.01^d^	0.34 ± 0.03^d^	0.78 ± 0.02^c^	1.12 ± 0.01^b^	1.55 ± 0.05^a^	*P* < 0.001

Values (mean ± *SE*) not sharing superscripts in the same row are significantly different (*P* < 0.05; One-way ANOVA). GLU: glucose; ALT: alanine aminotransferase; AST: aspartate aminotransferase

### Immunological and antioxidant/oxidant variables

The immune-related variables (LYZ, C3, SBA %, and antiprotease activity) of Nile tilapia subjected to acute MET toxicity are displayed in Table 4. These variables were significantly (*P* < 0.001) reduced by acute MET exposure (3–7.5 mg/L) relative to the control group. This reduction was in a level-dependent manner. However, no significant changes in these variables by 1.5 mg/L MET exposure. The acute MET exposure (1.5–7.5 mg/L) markedly (*P* < 0.001) decreased antioxidant variables (TAC, SOD, and GPx) and elevated the liver oxidant variable (MDA) compared to the control group (Table 4).

**Table 4:** Impact of various concentrations of metiram (MET) exposure for 96-hour on immunological and antioxidant/oxidant variables of Nile tilapia

Parameters	Concentration of MET (mg/L)						*P*-value
	0	1.5	3	4.5	6	7.5	
**Immunological variables**							
LYZ (ng/mL)	9.48 ± 0.28^a^	9.12 ± 0.06^a^	6.67 ± 0.15^b^	3.82 ± 0.05^c^	1.80 ± 0.08^d^	0.80 ± 0.05^e^	*P* < 0.001
C3 (mg/dL)	36.58 ± 0.24^a^	35.68 ± 0.97^a^	27.92 ± 0.18^b^	23.35 ± 0.20^c^	16.54 ± 0.54^d^	11.55 ± 0.20^e^	*P* < 0.001
SBA (%)	32.95 ± 0.17^a^	31.90 ± 0.31^a^	24.54 ± 0.17^b^	16.51 ± 0.22^c^	9.51 ± 0.16^d^	4.43 ± 0.18^e^	*P* < 0.001
Antiprotease (ng/mL)	28.28 ± 0.37^a^	27.85 ± 0.38^a^	15.66 ± 0.25^b^	12.91 ± 0.09^c^	3.54 ± 0.14^d^	1.20 ± 0.03^e^	*P* < 0.001
**Hepatic antioxidant/oxidant variables**							
TAC (ng/mg)	13.85 ± 0.08^a^	9.45 ± 0.26^b^	7.12 ± 0.07^c^	5.16 ± 0.10^d^	2.54 ± 0.19^e^	1.46 ± 0.03^f^	*P* < 0.001
SOD (U/mg)	218.70 ± 10.79^a^	192.80 ± 1.27^b^	136.12 ± 2.24^c^	105.39 ± 2.66^d^	73.28 ± 1.57^e^	33.06 ± 1.12^f^	*P* < 0.001
GPx (U /mg)	63.98 ± 0.33^a^	47.94 ± 0.40^b^	34.80 ± 0.60^c^	20.59 ± 0.25^d^	14.97 ± 0.29^e^	4.60 ± 0.26^f^	*P* < 0.001
MDA (nmol/mg)	0.73 ± 0.09^f^	1.46 ± 0.24^e^	8.27 ± 0.13^d^	15.90 ± 0.45^c^	26.19 ± 0.11^b^	39.30 ± 0.28^a^	*P* < 0.001

Values (mean ± *SE*) not sharing superscripts in the same row are significantly different (*P* < 0.05; One-way ANOVA). LYZ: lysozyme; C3: complement 3; SBA: serum bactericidal activity; TAC: total antioxidant capacity; SOD: superoxide dismutase; GPx: glutathione peroxidase; MDA: malondialdehyde

### Neuro-related variables

**Fig. 3:**
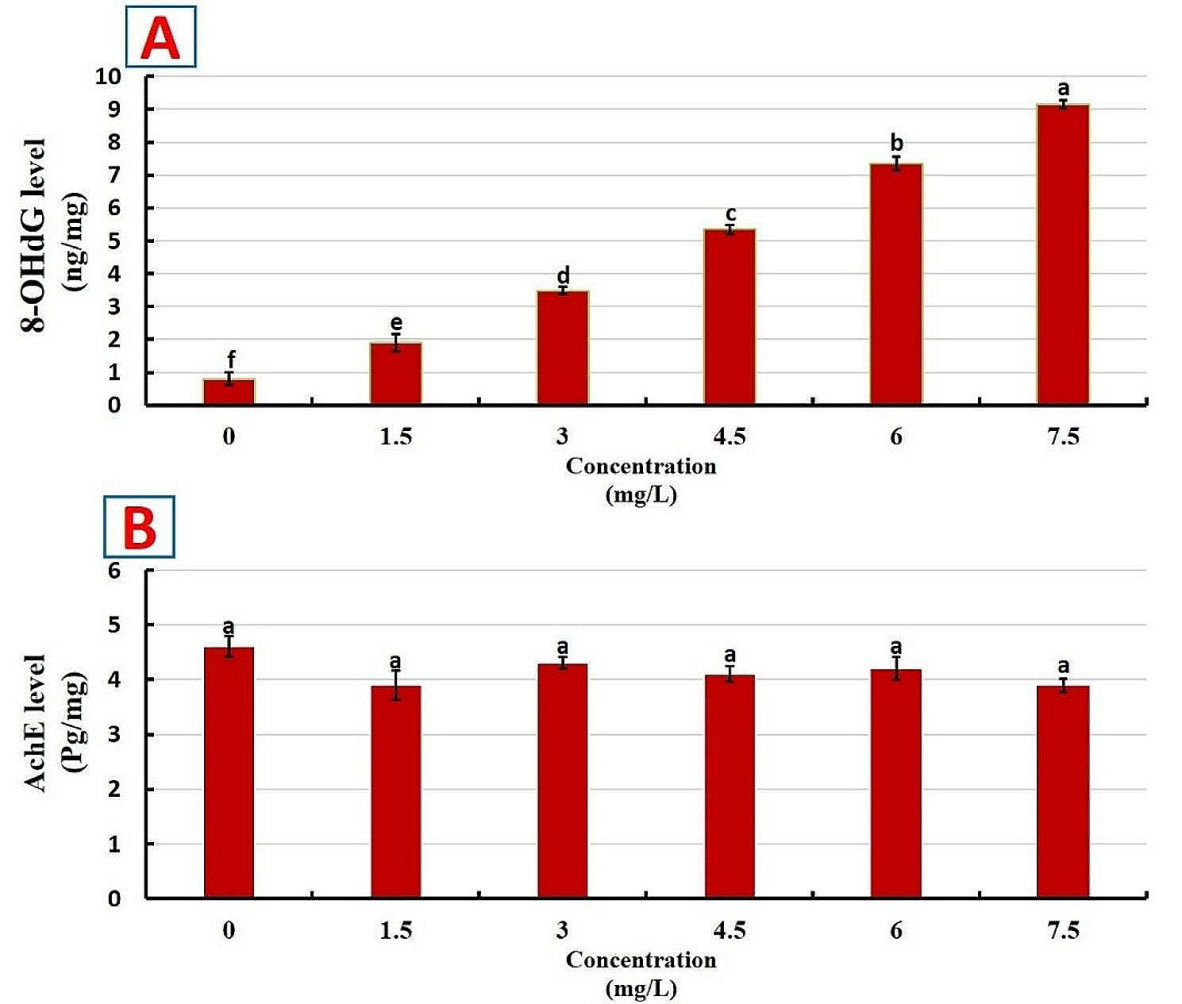
Impact of various concentrations of metiram (MET) exposure for 96-hour on neuro-related variables of Nile tilapia. **A** 8-hydroxy-2-deoxyguanosine (8-OHdG; *P* < 0.001). **B** Acetylcholine esterase (AchE; *P* = 0.54). Bars (mean ± *SE*) are not sharing superscripts significantly different (*P* < 0.05; One-way ANOVA)

The level of 8-OHdG (Fig. 3A) and AchE (Fig. 3B) as neuro-related biomarkers of Nile tilapia subjected to acute MET toxicity is shown in Fig. 3. The 8-OHdG showed a substantial (*P* < 0.001) increase by acute MET exposure (1.5–7.5 mg/L) in a level-dependent manner. On the contrary, MET exposure did not alter the AchE level.

## Discussion

Aquatic bodies are exposed to contaminants due to the extensive use of agrochemicals in agricultural practices. To control a wide range of fungal diseases in field fruits, vegetables, and crops, fungicides are applied. Fungicides enter surface waterways through spray drift, seepage, and runoff contaminating aquatic habitats [8]. Fish health is significantly impacted by aquatic pollution, which makes it difficult for aquaculture to grow sustainably [48]. Such contaminants not only harm fish health, but they may also harm human health via the food chain [49]. Metiram (MET) is one of many potentially toxic fungicides that enter the environment and endanger fish health. Despite the wide range of MET applications in agricultural practices, there is a lack of studies about the toxicological impacts of this fungicide on a widely and significant culture fish (Nile tilapia) [21, 22]. Given this context, our study was designated to better understand the acute negative effects of MET by using Nile tilapia as a model.

Acute toxicity evaluation provides the first estimation of the toxic effects of newly emerging contaminants and can assist in establishing concentration thresholds for further research into sub-lethal impacts [50]. According to our findings, the 96-hour LC_50_ of MET in Nile tilapia was 3.77 mg/L. Similarly, the 96-hour LC_50_ of MET was 1.1 mg/L and 85 mg/L in rainbow trout and common carp [14, 22], and 0.0025 mg/L in zebrafish [21]. Susceptibility and physiological differences between fish species might explain the observed variations in the MET 96-hour LC_50_ values.

Fish behavior after toxicant exposure is a reliable indicator of their physiological state, making it a useful tool for analyzing the impact of aquatic pollutants [51]. The MET-exposed fish displayed increasing in anomalous behaviors such as surfacing, loss of equilibrium, abnormal swimming and movement, and laterality with a reduction in the aggressive behaviors. Similar clinical observations have been recorded in yabby crayfish (*Cherax destructor*) [52], Nile tilapia [53], and common carp [54] after acute exposure to other fungicides. These results may be related to the risks that MET poses to the vital physiological systems of fish. MET exposure may cause oxidative damage by releasing reactive oxygen species (ROS) [55] into the gills, which are the tissues that come into close contact with the aqueous pollutant, which could lead to respiratory impairment. The preference for the uppermost layer (surfacing) could be a result of respiratory stress from the higher oxygen demand induced by toxicants [56]. This was proved by the marked elevation in the damage marker (8-OHdG) in our findings. The 8-OHdG is a commonly used marker to evaluate the genotoxic effect of contaminants including pesticides [57]. Oxidative stress products (ROS) as hydroxyl radicals of pesticides attack primarily target DNA resulting in the formation of 8-OHdG indicating oxidative stress [58].

Also, the aberrant behaviors caused by MET exposure could be related to the release of its toxic metabolites (carbon disulfide and ETU) that may contribute to disrupting brain functions causing neurotoxicity [59, 60]. Additionally, Bjørling-Poulsen et al. [61] verified that MET can localize in brain tissue and interfere with glutamate vesicular transport inducing neurotoxicity and oxidative stress. In this regard, loss of equilibrium, spiraling, and lateral swimming are most likely caused by nervous system dysfunction [62].

When fish are under toxicant exposure, the hematological profile is a useful technique for evaluating health status. Toxic contaminants in aquatic bodies produce acute hematological abnormalities [63]. The hematological parameters (RBCs, Hct, and Hb) of Nile tilapia were lowered in this study by acute MET exposure (1.5–7.5 mg/L), meanwhile, the MCH and MCV increased. Comparable results were previously documented in Nile tilapia and African catfish (*Clarias gariepinus*) [64, 65] by other fungicides (mancozeb and atrazine). This means that MET-fungicide negatively impacted the hematological markers showing anemia indicated by decreased RBCs and Hb in this investigation. A significant reduction in Hb level could have adverse effects on oxygen transport to different tissues, perhaps slowing down metabolism and leading to respiratory stress. According to a prior study [66], pesticides exposure may induce suppression of RBC or Hb synthesis resulting in lower RBCs count and Hb level. Moreover, another cause of anemia was ROS from pesticides exposure that can damage RBCs and oxidize Hb molecules which in turn reduces its oxygen-carrying ability [67]. The possible explanation for reduced Hct % (percentage of RBC in circulating blood) in MET-exposed fish was hemolysis or shrinkage of RBCs or destruction of hematopoietic tissues [68]. Additionally, the higher MCV value may be related to an increase in RBC size which has been observed in pesticide-exposed fish [69].

Acute exposure of fish to different toxicants including pesticides impacts the physiological state and stress biomarkers [19]. In fish, the accurate biomarkers of acute stress include blood cortisol, catecholamines, especially nor-epinephrine, and GLU [70, 71]. Herein, we observed significant changes in terms of increases in nor-epinephrine, cortisol, and GLU values in MET-exposed fish. These changes coincided with an increase in MET concentration, suggesting a strong stress reaction. According to Srivastava and Singh [72], there is also a possibility that the increase in GLU caused by MET exposure is due to an increase in gluconeogenesis in response to energetic demands during stress. Our findings align with recent research on Nile tilapia [64, 73] which demonstrated that stress was manifested as an increase in cortisol levels following Nile tilapia exposure to acute toxicity of other fungicides (96-hour).

The study evaluated hepato-renal markers (ALT, AST, and creatinine) and reported that there was an increase in response to acute MET exposure (4.5–7.5 mg/L). These findings suggested that exposure to MET severely harmed and impacted the hepatic and renal tissues by its ROS leading to dysfunction, which released these markers in the bloodstream. Comparable outcomes [74, 75] were noted in albino mice and rats following MET exposure.

WBCs are a marker of both immunological variables and physiological evidence of fish health. LYZ, C3, SBA, and antiprotease play important roles in fish humoral non-specific defense mechanisms against pathogens [76–78]. Water toxicants can alter humoral and cellular immunity resulting in a variety of deleterious effects on the fish body [79, 80]. This study looked at immune function biomarkers (WBCs and their differential count, LYZ, C3, SBA, and antiprotease) which were declined by acute MET exposure reflecting immuno-depression. By lowering the cytotoxic activity of natural killer cells and modifying T lymphocyte function, the immuno-depressive effect of MET was verified [81]. Other fungicides have similar effects on Nile tilapia [82] and Zebrafish [83]. The high level of cortisol that supports the stress situation caused by the MET may be the cause of the immunosuppressive effect that was observed in this investigation. According to Dunier [84] and Rehberger et al. [85], pollutant-induced immunosuppression might be viewed as a direct deleterious influence on immune cells or as a more indirect effect via corticoids (neuroendocrine system). Cortisol affects the immune system of fish by reducing the amount of circulating LYZ according to Guo and Dixon [86].

Oxidative stress is a major concern in the study of ecotoxicology. It is a condition in which the host body’s antioxidant defense mechanisms are out of balance with the generation of ROS following exposure to xenobiotics [87, 88]. Because it plays a vital role in detoxifying harmful substances and preparing metabolic products for breakdown, the liver often possesses the highest antioxidant defenses when compared to other organs [89, 90]. In this investigation, acute MET exposure in Nile tilapia resulted in higher lipid peroxides (MDA) levels and lower antioxidant defenses (TAC, SOD, and GPx) activity. These findings suggested a state of oxidative stress was brought on by acute MET exposure by raising the amount of ROS generation. Similar outcomes in zebrafish were documented [21]. Additionally, acute MET exposure induced various clinical signs (skin darkness, fin rot, and hemorrhages) and a reduction in fish survivability in a dose-dependent manner. The observed outcomes may be ascribed to reduced levels of antioxidant defense and elevated lipid peroxidation in the tissue, as indicated by elevated MDA levels. The reduced immunity due to acute MET exposure was as well.

Overall, the study’s findings revealed that acute exposure to MET caused toxicity in Nile tilapia, which was indicated by a lower survival rate, altered behavior, immunological, and neurological impairments, and stress in addition to oxidative damage that symbolized physiological disruption.

## Conclusion

This premier study determined the 96-hour LC_50_ of MET in Nile tilapia to be 3.77 mg/L. Fish exposed to various concentrations of MET exhibited significant behavioral and hepato-renal dysfunctions. Acute MET exposure elevated stress and brain indicator values and induced notable changes in immune-oxidant-related biomarkers. These findings reveal the toxic effects of MET. The impact of long-term MET exposure on the health of various fish species warrants further investigation.

## Data Availability

All data generated or analyzed during this study are included in this article.
